# Disintegration behavior and improvement mechanism of guar gum modified red clay in dry and wet cycle

**DOI:** 10.1371/journal.pone.0332742

**Published:** 2025-09-18

**Authors:** Binghui Zhang, Jiankun Hu, Ningshuan Jiang, Jicheng Xu, Donghua Han, Rui Duan, Jianrui Hu, Yanhua Xie

**Affiliations:** 1 Guangxi Key Laboratory of Geomechanics and Geotechnical Engineering, Guilin University of Technology, Guilin, China; 2 School of Civil Engineering and Transportation, South China University of Technology, Guangzhou, China; 3 Karamay Fucheng Oil Sand Resources Development Co., Ltd., Karamay, China; 4 Medical School, Shenzhen University, Shenzhen, China; 5 School of Energy Engineering and Building Environment, Guilin University of Aerospace Technology, Guilin, China; Jazan University College of Engineering, SAUDI ARABIA

## Abstract

As an improved agent, GG (Guar Gum) has been widely used in engineering reinforcement in recent years. To assess the potential of engineering applications in red clay areas, the study examined the disintegration behavior of red clay and the improvement effects of both pure and modified red clay containing 0.05%, 0.1%, 0.15%, 0.2%, and 0.25% GG under dry and wet cycle conditions. By quantifying the macroscopic mechanisms of GG’s impact on red clay and elucidating the microscopic mechanisms of GG’s improvement effects, the potential for engineering applications in red clay areas was confirmed. The experimental research results show that: (1) With an increase in GG dosage, the disintegration of red clay first decreases and then stabilizes, with the optimal improvement effect achieved at a GG dosage of 0.225%. (2) When the number of dry and wet cycles increases, the disintegration rate of GG-improved red clay increases less than that of unmodified soil. (3) Both macroscopic and microscopic improvement mechanisms show that GG can effectively improve the strength and stability of red clay by enhancing soil particles, improving particle grading, filling gaps, and forming GG kaolinite cementing bonds. In summary, GG has demonstrated great potential as an improved agent in engineering applications in areas with red clay. The research results enhance the understanding of the anti-disintegration behavior of red clay and provide a theoretical basis for improving soil engineering performance in areas with red clay.

## Introduction

Red clay is a distinct soil type commonly found in the southern regions of China. It is characterized by a high natural bearing capacity and cost-effective applicability [1]. However, its pronounced water sensitivity presents significant challenges for engineering projects in areas where red clay is prevalent [2,3]. This soil is prone to water absorption and expansion, as well as desiccation, which leads to shrinkage [4]. In subtropical and tropical monsoon climates, where dry and wet cycles are the norm, the soil’s strength can deteriorate significantly. Numerous studies have demonstrated that the cyclical wetting and drying processes cause cumulative damage within the soil, leading to a reduction in its irrecoverable strength and increased compressibility [5–8]. During drying, the soil shrinks and cracks as it loses moisture, while during rehydration, water rapidly infiltrates through the cracks, leading to soil softening, a reduction in strength, and even disintegration [9–13]. The degradation of soil structure caused by these dry and wet cycles [14], along with the expansion of cracks and eventual disintegration, are primary contributors to engineering failures such as roadbed instability, slope failure, and even collapse. These issues result in significant losses to both life and property [15]. Therefore, developing effective methods to mitigate the performance deterioration of red clay during dry and wet cycles is crucial.

In engineering practices, additive enhancement methods are commonly favored. Traditional reinforcing agents like cement and lime have been extensively utilized in numerous projects due to their ease of construction, accessibility, and significant reinforcement outcomes [16,17]. However, these conventional materials exhibit notable drawbacks. Primarily, the production of cement and lime generates substantial greenhouse gas emissions, depletes non-renewable resources, and poses a severe risk to the global ecological environment [18]. Additionally, soils treated with cement or lime tend to become brittle over time, leading to cracks in the soil structure and diminishing the long-term stability and durability of projects [19]. Consequently, the pursuit of more sustainable and effective soil amendments to supplant traditional materials has emerged as a critical area of research within geotechnical engineering.

Due to its environmental friendliness, stable enhancement effects, and low cost, polymers have garnered significant attention and application from both domestic and international researchers in the field of composite soil improvement [20–23]. Among these polymers, GG—a natural polymer extracted from guar bean seeds [24]—forms a highly viscous hydrogel network upon hydration. This network effectively fills soil pores and binds soil particles, thereby improving soil compactness and significantly reducing moisture permeability. GG is also fully degradable. It can be decomposed by microorganisms in the soil after fulfilling its engineering needs, and there is no environmental problem associated with chemical residue. In addition, in terms of cost sustainability, only a minimal amount of GG is required to achieve a good improvement effect. Kumar [25] focuses on three commonly used polymers and finds that GG can enhance shear strength, reduce permeability and improve soil stability, making the soil more resistant to wetting and drying processes. It also discusses the advantages of biopolymers such as GG in reducing greenhouse gas emissions. Zhang [26] used a variety of biopolymers to treat hot coffee soil, and evaluated the mechanical behavior and erosion resistance of biopolymers in soil samples treated with unbounded compressive strength test, split tensile test, triaxial test and pocket erosion instrument test. The results show that GG can significantly improve the unbounded compressive and tensile strength of the soil. Lei [27] used an improved device to study the permeability of bentonite with GG. According to detection indicators such as water effluent, pore water pressure, and conductivity, the water conductivity and central infiltration zone of the filter cake were studied. Combining these results with those from SEM, the research showed that a bentonite slurry containing GG can immediately form an external filter cake with a permeability zone. With a relatively short excavation distance, the excavated external filter cake once again forms a denser bentonite flake network, which can quickly stabilize the mud and drive the shield tunnel excavation surface. Hamza [28] conducted a comprehensive study on the geotechnical engineering performance of stable expansion subgrade of GG biopolymer, and examined the effects of different GG contents (0%-5%) and different aging periods on the properties of problem expansive soil. The study found that with the increase of GG content and aging time, the mechanical properties of soil both show an increasing trend, and the load-bearing capacity of soil treated with GG maintains ductility while maintaining its load-bearing capacity. Xu [29] evaluated the performance of GG-modified soil in western Jilin through unconfined compressive strength tests, disintegration tests, matrix suction measurements, and permeability tests, demonstrating its positive effect on the improvement of dispersive soils. Banne [30] conducted a series of laboratory experiments and XRD analyses to explore the influence of GG content on the mechanical properties of lateritic subgrade soil, confirming its beneficial impact. Sujatha [31] observed that the strength of GG-treated soil increased over time; although the biopolymer was susceptible to degradation, the degradation was inhibited, resulting in continued strength gain over a 90-day period. Subramani [32] employed advanced characterization techniques—including X-ray diffraction, X-ray fluorescence, Fourier-transform infrared spectroscopy, and scanning electron microscopy—to systematically analyze the mineral composition, functional group alterations, and morphological features of GG-amended soil. The findings revealed that GG-treated mixtures exhibited excellent ion adsorption capacity and hold promise as sustainable liner materials for sanitary landfills. Collectively, these studies confirm the effectiveness of GG in enhancing the mechanical properties of soil.

Although the detrimental effects of dry and wet cycles on soil structure have been extensively studied, and the potential of GG as an eco-friendly stabilizing agent has been preliminarily confirmed, comprehensive research on enhancing the engineering properties of red clay under these conditions, particularly systematic investigations into its disintegration behavior, remains limited. This is essential for mitigating engineering disasters caused by water sensitivity in regions with red clay. Consequently, this paper focuses on employing GG to improve red clay by systematically studying and analyzing the disintegration characteristics and strength variations of the treated soil under dry and wet cycles. A series of disintegration and direct shear tests was conducted to quantitatively assess the effectiveness of GG in enhancing the resistance of red clay to disintegration. Additionally, by examining changes in macroscopic shear strength and conducting microstructure analysis, the underlying mechanisms by which GG mitigates the impact of dry and wet cycles were elucidated. This study contributes to a deeper understanding of the anti-disintegration properties of red clay and provides a theoretical foundation for managing risks associated with water-sensitive disasters.

## Materials and methods

### Test materials

**Fig 1:**
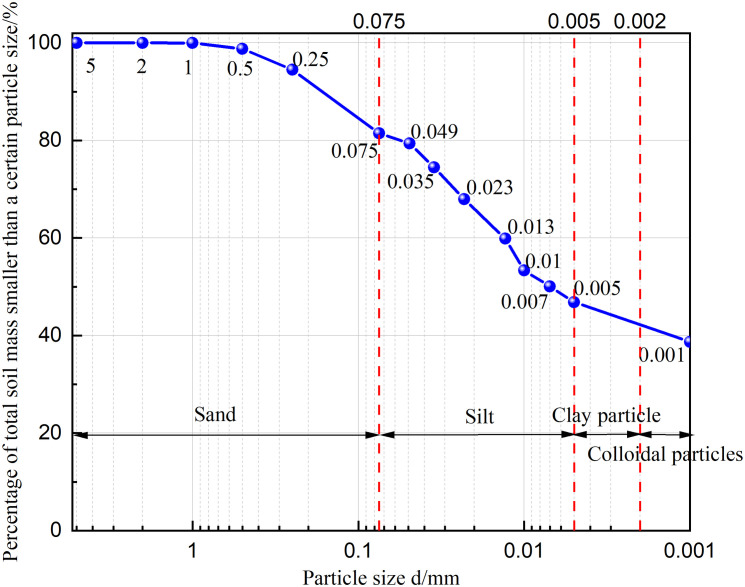
Red clay particle distribution curve.

**Fig 2:**
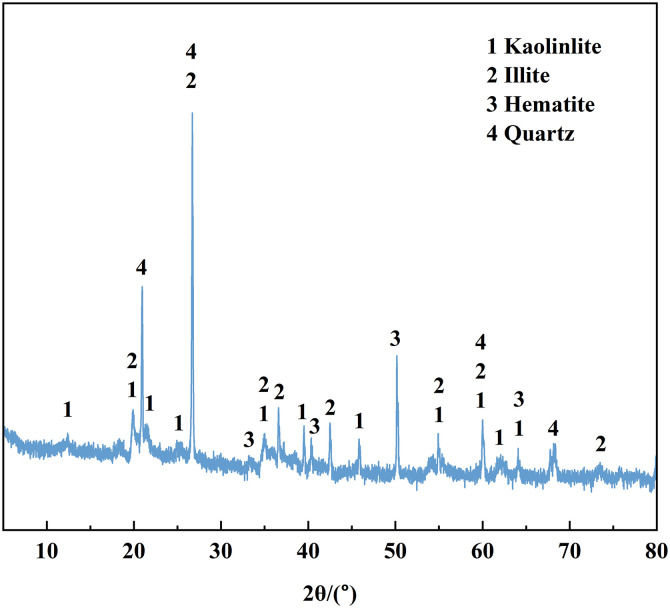
XRD diagram of red clay.

**Fig 3:**
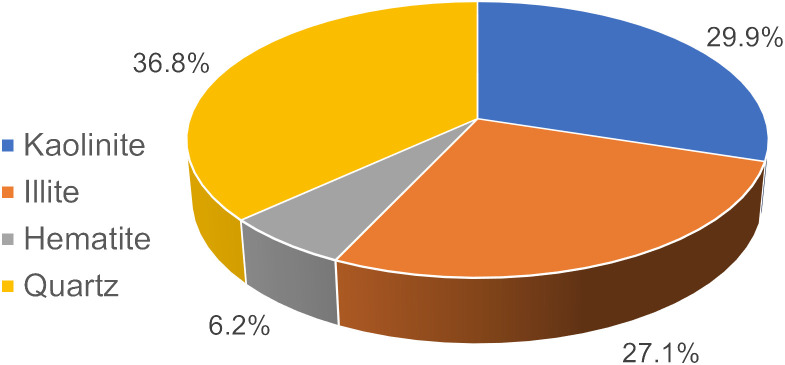
Composition ratio of red clay minerals.

The red clay used in the test was taken from the Yanshan campus of Guilin University of technology in Guilin, Guangxi. The red clay samples and experimental research in this study were approved by the school of civil engineering, Guilin University of technology. The soil samples were collected from the area of Yanshan campus of Guilin University of technology in Guilin, Guangxi, and did not cause environmental damage in the implementation process. After retrieving the soil sample, the basic physical properties of the soil sample were measured in detail in strict accordance with the provisions of the “Geotechnical Test Method Standard” (GB/T50123-2019), and the results are shown in Table 1. The particle analysis test was further conducted to understand the particle composition characteristics of red clay. The Red clay particle distribution curve was obtained (as shown in Fig 1). It can be seen from the figure that the clay mass fraction of <0.5 mm is as high as 98.8%, indicating that the red clay has an extremely high acceptable particle content. Fig 2 and 3 show the XRD patterns and mineral composition ratios of red clay, respectively. The characteristic diffraction peaks corresponding to each mineral can be seen from the figures. Through analysis of the spectra, it is found that the main mineral components are quartz, kaolinite, illite, and hematite, with proportions of 36.8%, 29.9%, 27.1%, and 6.2%, respectively.

**Table 1:** Basic physical properties of red clay.

Plastic limit $\omega_{L}/\%$	Liquid limit $\omega_{p}/\%$	Optimal moisture content ω/%	Maximum dry density $P_{d}/(g/cm^{3})$	Specific gravity
38.1%	61.8%	26.7%	1.5	2.74

**Fig 4:**
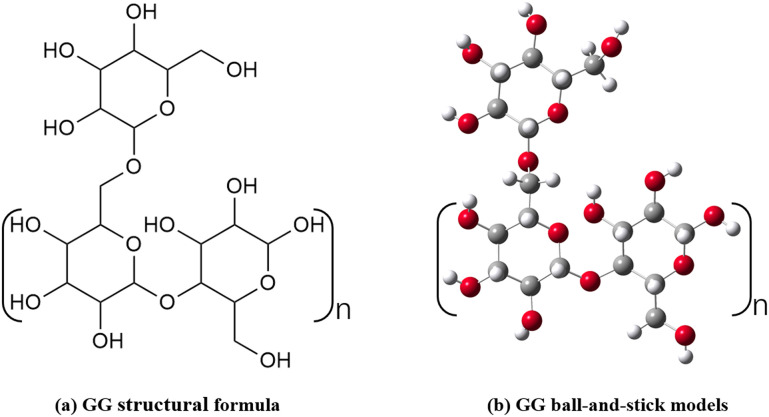
The structural formula and ball-and-stick models of GG.

During the test, GG was selected as the reinforcement material. GG used in this study is a food-grade powder provided by Beijing Guaran Technology Co., Ltd. The calibration purity of the product is  ≥ 98%. According to the data provided by the manufacturer, the viscosity of 1% (w/v) aqueous solution of GG at 25 ° C is 5000-5500mPa· s. The main chain of this natural polysaccharide is composed of (1 → 4) – linked β-D-mannose, and the branch chain is (1 → 6) – linked α-D-galactose. Its chemical structure, rich in hydroxyl, is the basis of its function as a soil conditioner. Its chemical formula is shown in Fig 4. GG is a neutral biopolymer extracted from guar bean seeds. It not only has excellent water solubility but is also one of the best-known natural polymers with the best water solubility. GG is in a light-yellow powder state. In addition to being easy to dissolve in water, it also has many advantages, such as high viscosity and biodegradability. Based on these advantages, GG has been widely used in many industrial fields, especially in food and pharmaceuticals. In this experiment, the potential of GG in the field of civil engineering material reinforcement will be explored, especially in red clay reinforcement.

### Dry and wet cycle scheme

**Fig 5:**
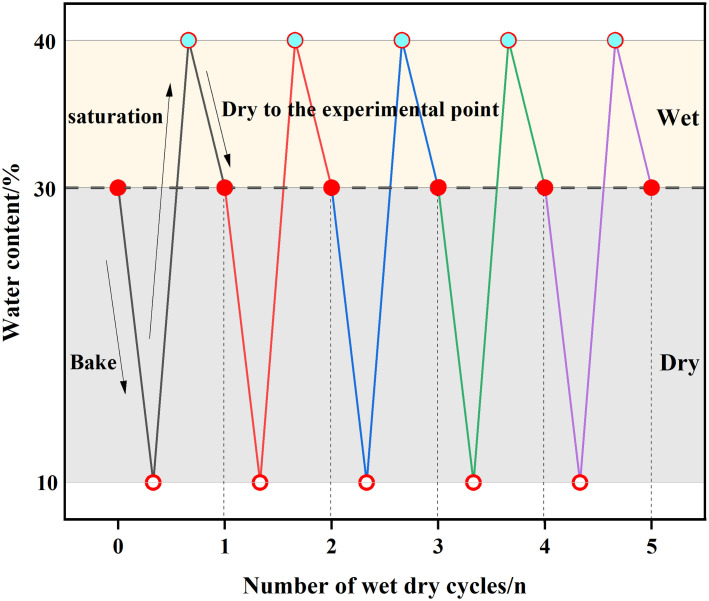
Dry and wet cycle diagram.

Guilin is located in the Guangxi Zhuang Autonomous Region in southern China and is at the intersection of the subtropical monsoon climate zone and the tropical monsoon climate. Its climatic characteristics are manifested as rain and heat during the same period, with distinct dry and wet seasons, especially in summer, with high temperatures and abundant precipitation. In order to simulate the local dry and wet cycle conditions accurately, this test adopts a process of drying first and then wetting. This cycle is achieved using an oven and a vacuum saturation cylinder. The temperature of the oven is set at 50 °C to simulate the natural drying temperature under the sunshine in this area. Specific operation rules of drying and wetting cycle: put the cured soil sample in the oven for drying, and test the moisture content several times during the drying process to ensure that the moisture content is controlled at the ideal value. When the moisture content drops to about 10%, end the drying process and humidify and saturate the sample, control the moisture content of the sample near the saturated moisture content, and then dry it again to 30% moisture content after saturation for 24h. During the drying process, test the moisture content several times to ensure accuracy. The dry and wet cycle diagram is shown in Fig 5. The ring cutter is retained during the cycle for subsequent direct shear tests.

### Sample preparation

The retrieved red clay was placed in a 101-2B electric-thermal constant temperature blower oven produced by Henan Qianwan Instrument Equipment Factory for drying. During the drying process, multiple weighing is used to ensure that the target mass is achieved. When the soil sample mass changes to less than 0.01g, it is considered to have reached a dehydrated state. Then, the dried red clay was crushed and passed through a 2 mm screen and then mixed evenly with the target amount of GG. As for GG content, based on the pre experiment (the content is 0.25%, 1%, 4% and 8%), we refer to the practice of relevant literatures such as Jin et al. [33], learn from the GG concentration range that can cause significant changes, refine the gradient of GG content on the basis of it, and also consider the economic feasibility of practical application. Here, the target content (i.e., the ratio of GG mass to dry soil mass m_G_/m_s_) is set to 0.05%, 0.1%, 0.15%, 0.2% and 0.25%. The numbering rules for the sample are shown in Table 2. Improved soil samples without dry and wet cycles are marked with $G_{k(k=0.05,0.1,0.15,0.2,0.25)}^{0}$, while modified soil samples that have undergone different dry and wet cycles (n = 1, 2, 3, 4, 5) are marked separately. For example, the sample number that has undergone one dry and wet cycle and has a GG content of 0.05% is $G_{0.05}^{1}$. If not specifically stated, the number of dry and wet cycles n is defaulted to 0−5 times, and the GG dosage k is defaulted to 0.05%−0.25%.

**Table 2:** Sample design and numbering of different dry and wet cycle times and GG dosage amounts.

	GG dosage k/%					
Dry and wet cycles n/time	0	0.05	0.1	0.15	0.2	0.25
0	$G_{0}^{0}$	$G_{0.05}^{0}$	$G_{0.1}^{0}$	$G_{0.15}^{0}$	$G_{0.2}^{0}$	$G_{0.25}^{0}$
1	$G_{0}^{1}$	$G_{0.05}^{1}$	$G_{0.1}^{1}$	$G_{0.15}^{1}$	$G_{0.2}^{1}$	$G_{0.25}^{1}$
2	$G_{0}^{2}$	$G_{0.05}^{2}$	$G_{0.1}^{2}$	$G_{0.15}^{2}$	$G_{0.2}^{2}$	$G_{0.25}^{2}$
3	$G_{0}^{3}$	$G_{0.05}^{3}$	$G_{0.1}^{3}$	$G_{0.15}^{3}$	$G_{0.2}^{3}$	$G_{0.25}^{3}$
4	$G_{0}^{4}$	$G_{0.05}^{4}$	$G_{0.1}^{4}$	$G_{0.15}^{4}$	$G_{0.2}^{4}$	$G_{0.25}^{4}$
5	$G_{0}^{5}$	$G_{0.05}^{5}$	$G_{0.1}^{5}$	$G_{0.15}^{5}$	$G_{0.2}^{5}$	$G_{0.25}^{5}$

Next, the moisture content of the mixed soil sample was adjusted to 30% using distilled water, and sealed measures were taken to place it in a moisturizing tank for 24 hours. After the maintenance is completed, the static pressure method is used to prepare direct shear samples and disintegrated samples with different GG dosages according to the specifications. The dimensions of the straight shear sample are ϕ 61.8 mm × 20 mm, while the dimensions of the disintegrated sample areϕ 79.8 mm × 20 mm.

### Test content

Various pre-ratioed samples were used for disintegration test, direct shear test and scanning electron microscope test according to the “Geotechnical Test Method Standard (GB/T50123-2019)”.

### Disintegration test

**Fig 6:**
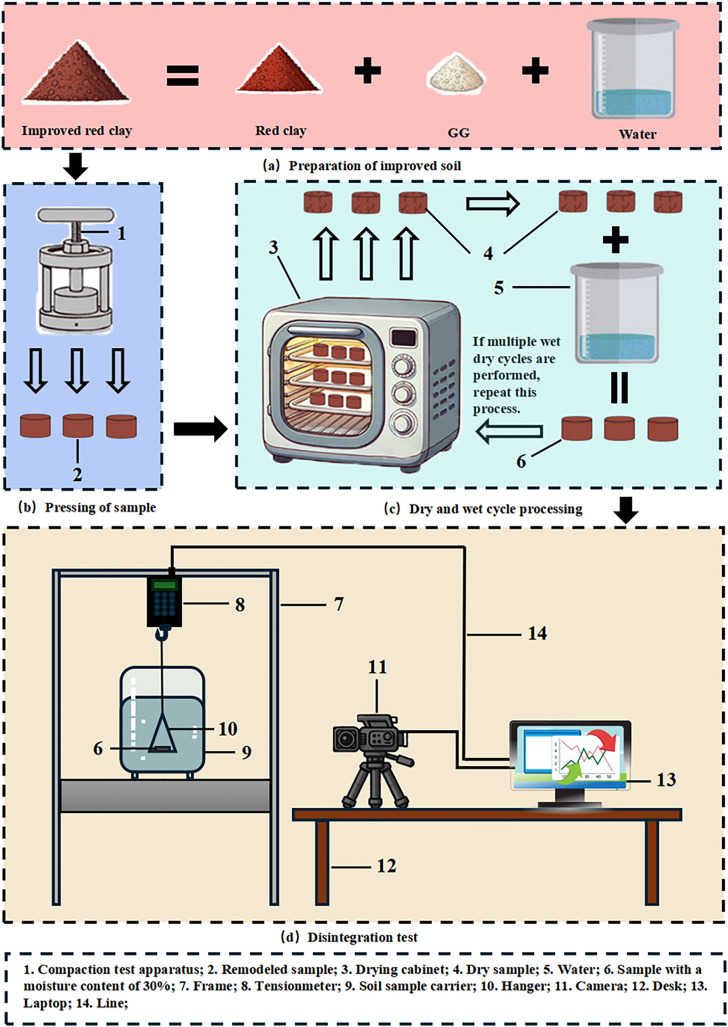
Sample preparation and disintegration test flow chart.

The self-made disintegration instrument is used in the disintegration test. The self-made disintegration instrument is mainly composed of a sample carrier, water tank, camera, push-pull meter and computer. Among them, the push-pull meter adopts the German NSCING push-pull meter, the specific model is SH-III-30N, which has passed the verification and certification of the national law meter, with a high-precision sensor, and the accuracy can reach 0.5%. As shown in Fig 6 (d), the flow chart is shown in Fig 6. During the whole process of the test, the temperature in the control room was 20°C, and the humidity in the room was 50%-60% to control the consistency of the external conditions. The significant advantages of this instrument are its simple structure, easy access to components, and the ability to monitor the disintegration process in real time. In the test, 86% compaction ring cutter samples were used, which were placed in a homemade disintegration instrument after undergoing different numbers of dry and wet cycles. Through a high-precision push-pull gauge, the quality changes during the disintegration process can be measured and recorded in-real time, which is accurately monitored by computer data acquisition software. When it is observed that there are no residual samples on the mesh plate or that the remaining samples no longer disintegrate significantly for a long time, and the reading of the push-pull gauge remains stable, the test is determined to be over.

After the test, the disintegration characteristics of the sample were quantitatively analyzed using the index parameter of disintegration (Equation 1). The disintegration amount can intuitively reflect the degree and characteristics of the sample, and can provide strong data support for subsequent research and analysis.


$$K_{t}=\left(\frac{H_{0}-H_{t}}{H_{0}}\right)\times 100\%,$$ (1)

Where $K_{t}$ is the disintegration amount of the sample at time t/%, $H_{0}$ is the reading of the push-pull force meter at the beginning of the test/kg, and $H_{t}$ is the reading of the push-pull force meter at time t/kg.

### Direct shear test

The instrument used in the direct shear test is the ZJ-type strain-controlled direct shear instrument (quad-coupled shear) manufactured by Nanjing Soil Instrument Co., Ltd. The test subjects are $G_{k(k=0,0.05,0.1,0.15,0.2,0.25)}^{n(n=0,1,2,3,4,5)}$, and the shear rate set in the test is 0.8 mm/min. When the shear displacement reaches 4 mm, it is considered to be the endpoint of the test; if no prominent peak is observed during this process, the shearing continues until the displacement reaches 6 mm. In the straight shear test, the shutdown displacement is uniformly set to 6 mm to ensure the integrity of the test and the accuracy of the data.

### SEM scanning electron microscopy test

To observe the microstructure characteristics of soil, the JSM-7800F field emission scanning electron microscope (SEM) was used for the experiment. The test object is $G_{k(k=0.25)}^{n(n=5)}$, and the sample is prepared using the fresh surface of the sample. First, the soil sample is cut into small pieces of about 1 cm^3^. After the ordinary soil sample is treated, it is dried at a low temperature in an oven at 40°C. For the modified soil sample of GG, in order to avoid drying and dehydration affecting the structure, the liquid nitrogen freeze-drying method is prepared by vacuum freezing. Before spraying gold, cut the sample and use the eraser to remove the surface-dispersed soil to expose the fresh surface. Then, the sample is cut down, and the fresh surface is facing up and firmly pasted on the metal base with conductive glue to prevent shaking during scanning and affecting imaging. Finally, the sample is sent to a small ion sputtering instrument for gold spraying, and the SEM test can be performed after completion.

## Results and analysis

### $G_{k(k=0,0.05,0.1,0.15,0.2,0.25)}^{0}$ disintegration phenomenon and analysis

**Fig 7:**
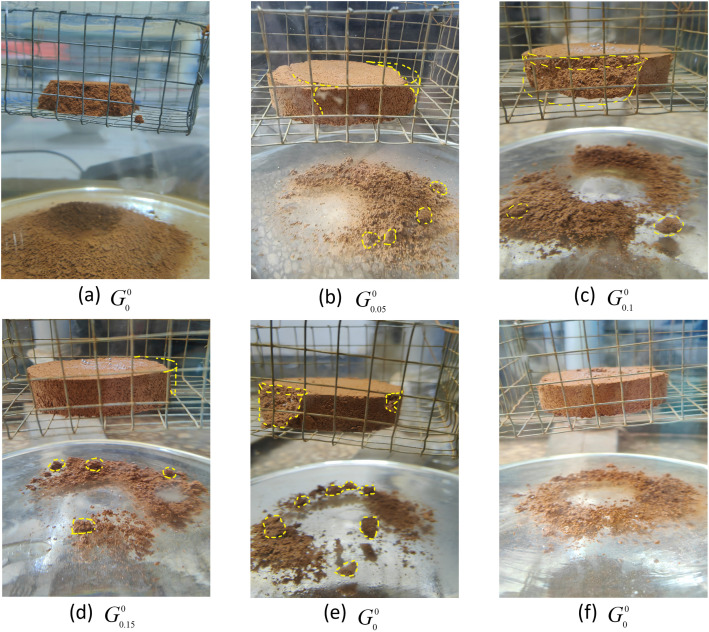
$G_{k(k=0,0.05,0.1,0.15,0.2,0.25)}^{0}$ stable disintegration and destruction pattern. (a) $G_{0}^{0}$ (b) $G_{0.05}^{0}$ (c) $G_{0.1}^{0}$ (d) $G_{0.15}^{0}$ (e) $G_{0.2}^{0}$ (f) $G_{0.25}^{0}$.

Fig 7 shows the disintegration phenomenon of samples with different GG dosages without drying and wet action. The untreated specimen (Fig 7(a)) displayed severe disintegration marked by yellowish effluent, foam formation, and massive particle detachment, culminating in irregular debris. Contrastingly, GG-modified specimens exhibited fundamentally altered behavior: water clarity improved substantially while suspended particulates diminished. Nevertheless, specimens with suboptimal GG content (Fig 7(b)) demonstrated persistent macroparticle shedding and crack propagation—this appears to suggest, albeit requiring microstructural verification, incomplete development of the cementation framework. Significantly, high-dosage GG specimens (Fig 7(f)) maintained structural coherence post-disintegration with refined detritus. This phenomenon likely stems from gel-phase materials effectively occluding soil voids, thereby disrupting what is commonly termed the “moisture intrusion pathway.”

**Fig 8:**
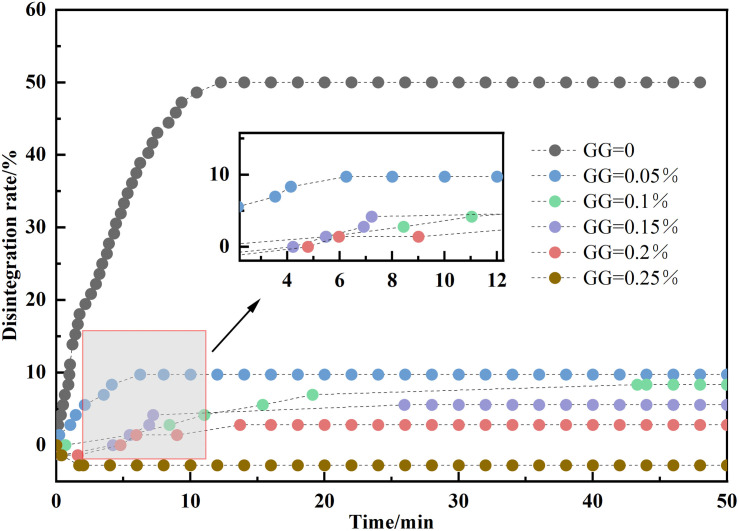
Time-disintegration rate curve at different GG dosage amounts.

The gradient of the disintegration rate curve serves as a direct indicator of disintegration velocity, where steeper gradients correspond to accelerated disintegration and shallower gradients denote decelerated processes. As delineated in Fig 8, three distinct disintegration phases were identified through temporal-rate correlation analysis: (1) An initial phase marked by rapid water permeation and gas emission, during which no measurable sample disintegration was observed; (2) An intermediate phase exhibiting pronounced curve gradient intensification. Notably, specimens with low GG content demonstrated not only accelerated disintegration but also exhibited discrete soil block detachment during this phase; (3) A terminal phase where curve gradients asymptotically approached zero, indicating attainment of disintegration equilibrium. Disintegration equilibrium was established when the curve gradient asymptotically approached zero in the terminal phase. $G_{0}^{0}$ reached a maximum disintegration rate of 50% within 12 minutes, while $G_{0.05}^{0}$,$G_{0.1}^{0}$,$G_{0.15}^{0}$,$G_{0.2}^{0}$, and $G_{0.25}^{0}$ reached their highest disintegration rates of 9.72%, 8.33%, 5.56%, 2.78%, and −2.78% after 6, 43, 26, 13, and 2 minutes, respectively. Of particular interest is the $G_{0.25}^{0}$ specimen manifesting anomalous −2.78% disintegration due to hygroscopic mass gain surpassing disintegration loss.

**Fig 9:**
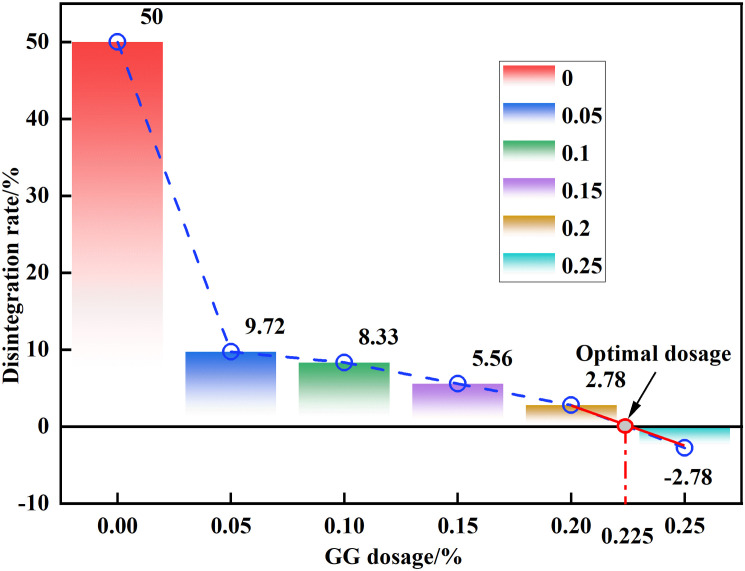
Disintegration rate-GG doping amount.

The relationship between disintegration rate and GG concentration is depicted in Fig 9. Experimental data indicate that as GG content continuously increases, the resistance to disintegration demonstrates an exponential growth trend. The observed phenomenon suggests that, prior to microstructure validation, the resistance may stem from the physical filling of soil pores by GG particles, enhanced cohesion between soil particles, and the formation of a bonding network, which together could suppress the hydraulic fracturing mechanism. It is noteworthy that at a GG dosage of 0.225%, the critical threshold, the disintegration rate ceases, marking this as the optimal dosage. At this juncture, pore filling and bond strengthening achieve a dynamic equilibrium. This is highly consistent with the results of many studies on soil improvement by biopolymers. For example, Rong [34] used GG to improve loess and found that when the amount of GG was 0.2%, the sample almost did not disintegrate. In addition, compared with traditional reinforcement agents (such as cement), this optimal dosage is more competitive. It not only requires less dosage, but also can significantly reduce the damage to the environment. At the same time, the low dose is also easy for the construction operation of the actual project.

### Disintegration phenomenon of GG improved red clay under dry and wet cycle and analysis

**Fig 10:**
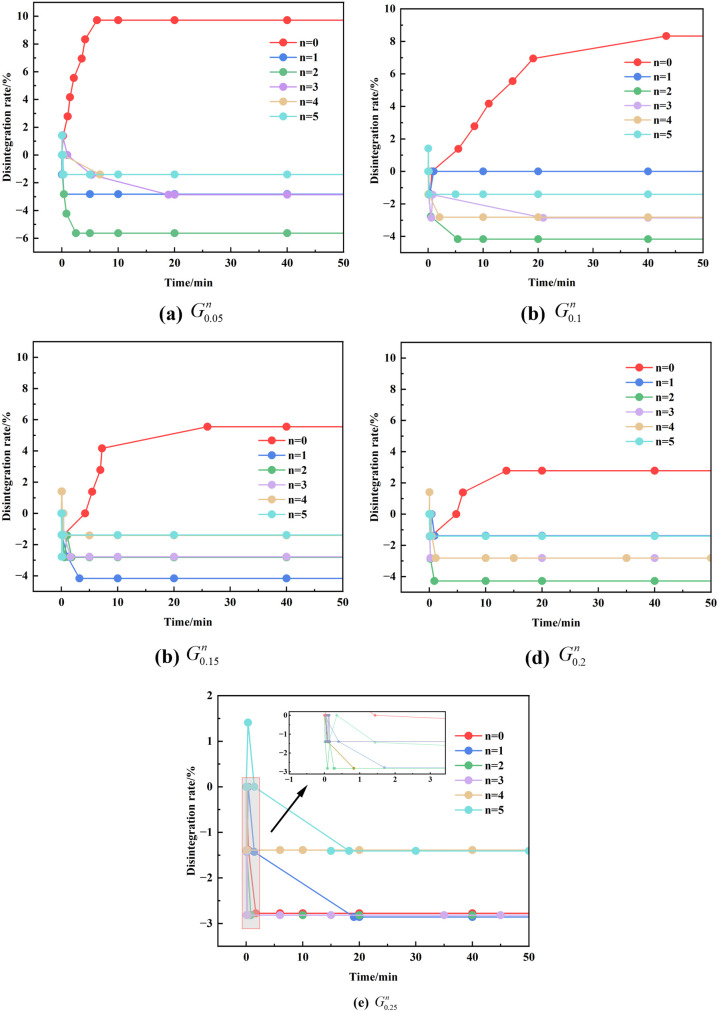
$G_{k}^{n}$ under the action of dry and wet cycles. (a) $G_{0.05}^{n}$ (b) $G_{0.1}^{n}$ (c) $G_{0.15}^{n}$ (d) $G_{0.2}^{n}$ (e) $G_{0.25}^{n}$.

Fig 10 illustrates the relationship between time and disintegration rate under alternating dry and wet cycles. It can be seen that the disintegration rate of all samples rapidly decreases to negative values when first immersed in water and then gradually rises or continues to decline until it stabilizes. This occurs as dry samples rapidly absorb water upon exposure, resulting in an increase in their overall mass. In the initial dry and wet cycle, samples absorb the most water, leading to a significant weight increase. During subsequent cycles, repeated soaking and drying result in the loss of water and soil particles from the samples, which causes the disintegration rate to slowly recover after reaching its nadir; however, the overall value remains negative due to continued water absorption.

As illustrated in Fig 10 (e), when (n = 0, 1, 2, 3, 4, 5), the curve patterns of the $G_{0.25}^{n}$ samples are mainly consistent. In the absence of humidification (0 cycles), the samples rapidly absorbed water upon immersion, resulting in a swift decline in the disintegration rate to a negative value, which subsequently stabilized. After undergoing five dry and wet cycles, the disintegration rate reached its peak at 1.4%.

Fig 10 also reveals an important trend: under the influence of alternating dry and wet conditions, as the amount of GG incorporation increases, the extent of disintegration decreases significantly, and the disintegration rate becomes negative. This indicates that the incorporation of GG effectively mitigates the cumulative damage caused by repeated dry and wet cycles, substantially reducing the disintegration rate of red clay.

### Improvement mechanism of GG improved red clay

#### Macro-improvement mechanism based on shear strength.

**Fig 11:**
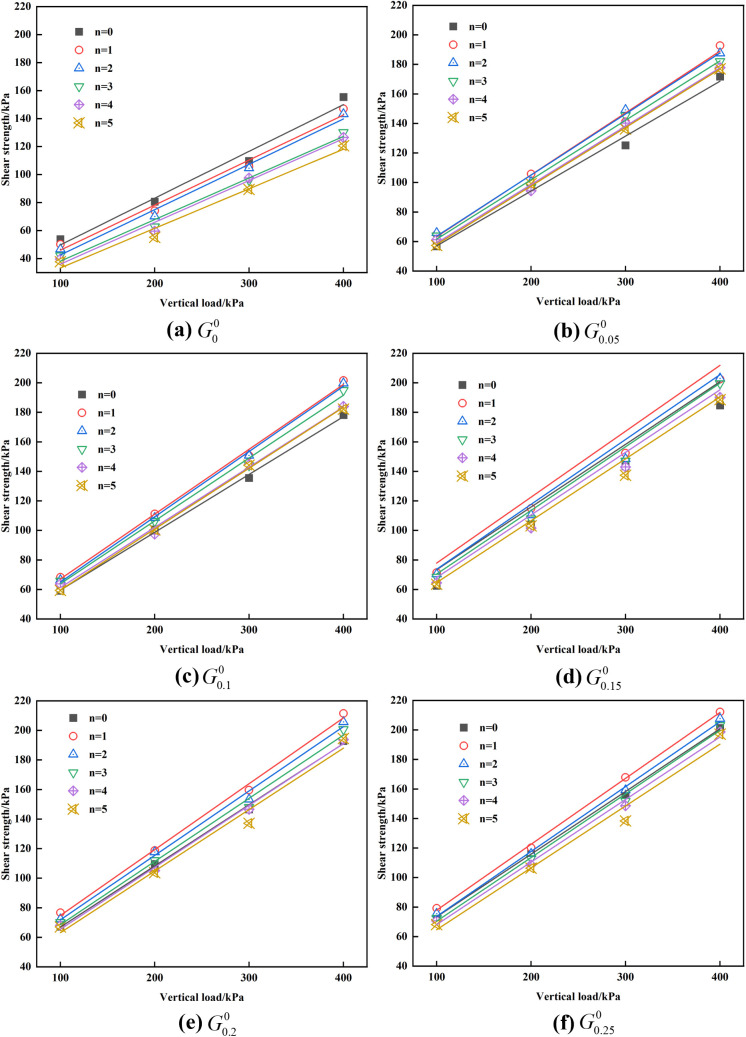
The relationship between vertical load and shear strength under different GG content and different cycle times. (a) $G_{0}^{n}$ (b) $G_{0.05}^{n}$ (c) $G_{0.1}^{n}$ (d) $G_{0.15}^{n}$ (e) $G_{0.2}^{n}$ (f) $G_{0.25}^{n}$.

Shear strength is the ability of soil to resist shear damage and is one of the important indicators to measure the nature of soil engineering. Fig 11 illustrates the shear strength relationship curves of sample $G_{k(k=0,0.05,0.1,0.15,0.2,0.25)}^{n(n=0,1,2,3,4,5)}$ under vertical pressures of 100 kPa, 200 kPa, 300 kPa, and 400 kPa. The results demonstrate that the dry and wet cycle significantly affects shear strength. Following the dry and wet cycle, the shear strength shows a decreasing trend. Specifically, after five dry and wet cycles, the shear strength of sample $G_{0}^{n(n=0,1,2,3,4,5)}$ decreased by 29.8%, 31.5%, 18.5%, and 22.2% under vertical pressures of 100 kPa, 200 kPa, 300 kPa, and 400 kPa, respectively, with a reduction range of 18.5% to 31.5%.

Moreover, incorporating GG significantly enhances red clay’s shear strength. Under the same vertical pressures, the shear strength of sample $G_{0.05}^{5}$ increased by 51.7% at 100 kPa, 79.8% at 200 kPa, 52.3% at 300 kPa, and 46.8% at 400 kPa compared to sample $G_{0}^{5}$, with the increase ranging from 46.8% to 79.8%. Further comparison of samples with varying GG contents reveals a positive correlation between the shear strength and GG content. As the GG content increases, the shear strength of red clay gradually increases.

In order to more accurately quantify the improvement effect of GG on the shear strength of red clay under dry and wet cycles, statistical analysis is performed on the shear strength data of each group by using mean value (Equation 2), standard deviation (Equation 3) and 95% confidence interval (Equation 4). The mean value, standard deviation (SD) and 95% confidence interval (CI) of shear strength of samples under 0–5 dry and wet cycles and different vertical pressures are shown in Table 3.

**Table 3:** Statistical analysis table of shear strength data.

Group	Average shear strength(kPa)	Standard deviation	95%Confidence interval (kPa)
$G_{0}^{n}$	87.44	36.67	[71.95,102.92]
$G_{0.05}^{n}$	120.42	46.05	[100.97,139.86]
$G_{0.1}^{n}$	125.72	48.35	[105.31,146.14]
$G_{0.15}^{n}$	128.44	48.58	[107.93,148.95]
$G_{0.2}^{n}$	132.41	49.17	[111.65,153.17]
$G_{0.25}^{n}$	135.95	49.64	[114.99,156.91]


$$\bar{x}=\frac{1}{n}\sum_{i=1}^{n}x_{i},$$ (2)

In formula (2), $\bar{x}$ is the shear strength, $x_{i}$ is the shear strength data point, and n is the total number of samples in each group, i.e., 24.


$$s=\sqrt{\frac{1}{n-1}\sum_{i=1}^{n}{\left(x_{i}-\bar{x}\right)}^{2}}$$ (3)

$$CI=\bar{x}\pm t_{\alpha /2,n-1}\cdot \frac{s}{\sqrt{n}}$$ (4)

In formula (3) and (4), n is the total number of samples in each group, i.e., 24, $t_{\alpha /2.n-1}$ is the critical value of t distribution with degree of freedom n-1 = 23, t ≈ 2.069 according to the table, s is the standard deviation of samples and $\bar{x}$ is the value of samples.

The standard deviation reflects the dispersion of shear strength values under different dry and wet cycles and vertical pressures. It can be seen from Table 3 that SD values of all groups are large, which is also consistent with the significant influence of dry and wet cycles and vertical pressures on shear strength values in the test. By comparing the confidence intervals of different groups, it can be found that the CI of $G_{0.05}^{n}$ is [100.97, 139.86], the confidence interval of $G_{0}^{n}$ is [71.95, 102.92], the upper limit of CI of $G_{0}^{n}$ and the lower limit of $G_{0.05}^{n}$ almost do not overlap, and there is no overlap with higher GG dosage groups such as $G_{0.1}^{n}$ and $G_{0.15}^{n}$. This indicates that the incorporation of GG does effectively increase the intensity, resulting in an improvement in the data obtained from a statistical analysis perspective.

GG improver, as an effective soil stabilizer, primarily enhances the shear strength of red clay through a distinct physicochemical mechanism. (1) The introduction of GG alters the chemical composition of red clay. GG molecules, rich in functional groups such as hydroxyl and carboxyl, interact with metal ions in the soil to form chemical bonds. This reaction not only strengthens the bonds between soil particles but also promotes particle agglomeration, thereby enhancing the soil’s shear strength. (2) The addition of GG significantly improves the particle distribution of red clay. Given the high acceptable content and small inter-particle gaps in red clay, it tends to form a dense structure. The long chains of GG molecules bridge these particles, creating a spatial network that enhances inter-particle connectivity. This structural modification increases the soil’s resistance to particle displacement under shear stress, thus boosting shear strength. (3) The water retention properties of GG also critically influence the shear strength of red clay. The moisture content in red clay fluctuates with wetting and drying cycles, but GG forms a viscous gel upon water contact. This gel fills soil voids and tightens particle connections, improving soil cohesion. Additionally, its moisture retention mitigates the volumetric changes during soil drying, increasing shear resistance.

### Micro-improvement mechanism based on SEM images

**Fig 12:**
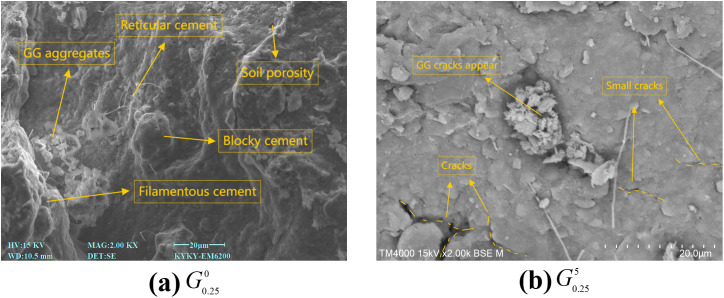
SEM image. (a) $G_{0.25}^{0}$ (b) $G_{0.25}^{5}$.

Fig 12 presents a scanning electron microscope image of the sample $G_{0.25}^{0}$, which has not been subjected to dry and wet effects. As depicted in Fig. 12(a), the sample’s surface is smooth, devoid of noticeable pores or cracks, and maintains an intact overall structure. GG is closely combined with soil particles to form a large number of GG kaolinite and other cementation bonds, which are closely related to the chemical structure of GG. According to the GG structure formula and the bat model, these molecular chains contain a large number of hydroxyl groups. At the same time, because the main minerals of red clay include kaolinite, illite and hematite, these crystal surfaces are exposed with a large number of oxygen atoms and hydroxyl groups. The hydroxyl groups on the GG molecular chain react with the oxygen atoms or hydroxyl groups on the surface of these mineral components to form these hydrogen bonds, which is what we call cementation bonds, which is highly consistent with the research conclusion of Bane et al. [30], these hydrogen bonds connect adjacent soil particles to form a filiform and network structure, while the soil wrapped by GG the particles present a massive structure, which effectively fills the pore space. This finding is also consistent with the research of many scholars. For example, Soldo [35] found that after adding GG, GG and fine particles formed a dense structure attached to large particles. Bonal [36] confirmed by SEM micrograph that bauxite residue particles can be connected by GG, thus improving the shear strength. It is certain that this structure significantly enhances the cohesion of soil, reduces the disintegration rate, and improves the shear resistance, so that the soil can better resist potential damage.

After undergoing five dry and wet cycles, the SEM image of $G_{0.25}^{5}$, depicted in Fig. 12(b) shows that a substantial amount of filamentous, mesh, and blocky cements are preserved on the sample’s surface. Minor breakage and tiny cracks are visible, and the soil particles encapsulated in GG are slightly loosened. Nevertheless, the distribution of these structures, both internally and on the soil’s surface, effectively impedes water penetration, mitigates moisture infiltration, and decreases the cracking caused by the water absorption and expansion of soil particles. Consequently, despite multiple dry and wet cycles, the soil sample’s surface remains intact, and its overall structure is stable, with no significant pores or continuous cracks observed. At the same time, observing the surface of GG modified sample, it can be seen that the surface of the sample is covered with a large number of films formed by cemented bonds, which bridge the tiny pores and cracks on the surface of the sample, directly leading to a significant increase in the shear strength of the sample, and there are still a large number of cemented bonds after several dry and wet cycles, which is consistent with the results of disintegration and shear tests, further proving the stability and durability of the sample under the action of dry and wet cycles.

**Fig 13:**
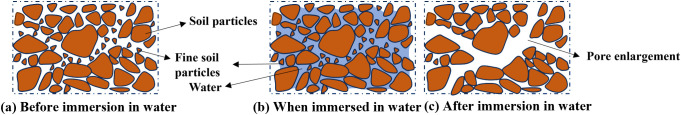
$G_{0}^{n(n=0,1,2,3,4,5)}$ disintegration microstructure change diagram.

Fig 13 illustrates the microstructural changes in the disintegration of $G_{0}^{n(n=0,1,2,3,4,5)}$. Fig 13(a) demonstrates that the soil, containing numerous pores, rapidly absorbs water upon immersion, filling its internal voids. As depicted in Fig 13(b) and 13(c), the progression of the dry and wet cycles results in the loss of fine-grained soil with water, which enhances the penetration of pores within the soil. Consequently, small pores gradually enlarge into larger pores, thereby accelerating the rate of water infiltration. Simultaneously, water infiltration diminishes the binding force between soil particles, leading to their separation and detachment. This process continues layer by layer until the soil disintegrates and stabilizes.

**Fig 14:**
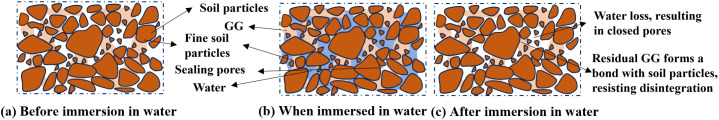
$G_{k(k=0,0.05,0.1,0.15,0.2,0.25)}^{n(n=0,1,2,3,4,5)}$ disintegration microstructure change diagram.

The changes in the disintegration microstructure after the addition of GG are shown in Fig 14. Due to the incorporation of GG, when immersed in water (Fig 14(b)), the hydroxyl groups (—OH) on its linear chain interact with water molecules to form hydrogen bonds, resulting in the formation of a hydrogel. This hydrogel not only creates cementitious bonds between soil particles, strengthening the soil skeleton, but also fills pore spaces, making the soil denser and reducing moisture infiltration. After sufficient immersion (Fig 14(c)), GG and soil particles collectively surround specific pores, forming a closed space that further weakens water infiltration. At the same time, the hydrogel enhances the cohesion between soil particles, reduces the loss of fine-grained soil, and decreases the disintegration rate. During the drying phase, the cohesion between the red clay particles is significantly reduced due to moisture evaporation, which leads to crack formation and a decrease in shear strength. However, the presence of GG ensures the cementitious strength of the soil. GG effectively bonds the surrounding soil particles, improves soil water retention, reduces the formation of new pores, and inhibits the expansion of existing cracks, thereby maintaining the overall integrity of the soil sample.

Detailed analysis of SEM images shows that the GG modifier significantly enhances the microstructure of red clay. These microscopic alterations underpin the observed macroscopic performance improvements. The GG modifier comprehensively enhances red clay by altering its chemical composition, optimizing particle distribution, and enhancing pore characteristics. This establishes a robust foundation for its extensive application in engineering practices.

## Conclusions

This study employs GG as an enhancement agent to improve the physical properties of red clay by incorporating varying proportions of GG. It extensively examines the disintegration behavior of GG-modified red clay under both dry and wet conditions and explores the underlying mechanisms of its improvement. The primary conclusions drawn are as follows:

1. The disintegration behavior of red clay significantly improved after adding GG, particularly under the influence of dry and wet cycles. As the dosage of GG increased, the disintegration rate of the red clay initially decreased and subsequently stabilized. At a doping ratio of 0.225%, the disintegration rate was notably reduced to 0%, demonstrating exceptional resistance to disintegration.
2. The dry and wet cycling process significantly affects the disintegration behavior of modified red clay. As the number of dry and wet cycles increases, the disintegration rate of improved and unimproved red clay rises. However, the disintegration rate of the modified soil increases at a slower rate than that of the unmodified soil, further demonstrating the effectiveness of GG modification.
3. Based on the analysis of the macro-mechanism for improving shear strength, adding GG enhances the bonding between soil particles through chemical interactions. It also improves particle gradation, forming a denser soil structure, and fills voids by retaining water, thereby reducing volume changes caused by drying. These effects collectively lead to a significant improvement in both the shear strength and resistance to the disintegration of red clay.
4. The micro improvement mechanism based on SEM images shows that the GG modifier enhances the cohesion and structural stability between soil particles by forming GG kaolinite bonds. At the same time, GG can also form hydrogel to fill the pores in filamentous, reticular and blocky forms, strengthen the soil skeleton, reduce the loss of fine particles, and reduce the disintegration rate.
5. The research results are of great significance for the areas dominated by red clay. Compared with the traditional reinforcement agent, GG improved method provides a feasible, effective and environmentally friendly alternative. In highway construction, GG improvement method can be used to alleviate the settlement and cracking problems caused by repeated water fluctuations. In dealing with the problem of slope stability, applying GG improved soil layer on the slope can effectively enhance the shear strength and reduce the landslide problem. In the rainy season with huge rainfall, GG improved method can also maintain good effect.
6. Although the GG improved method has various advantages, its limitations can not be ignored. Specifically, GG is susceptible to microbial degradation, and its long-term stability still needs further evaluation. In addition, the unit cost of GG is higher than that of cement. When considering the economy of the project, it is necessary to analyze its construction cost and maintenance cost.

The study of the disintegration behavior of GG-improved red clay under dry and wet conditions not only reveals the underlying improvement mechanisms but also confirms its potential for engineering applications in red clay regions. This research enhances the understanding of the anti-disintegration behavior of red clay and provides a theoretical foundation for engineering construction and disaster mitigation in areas with red clay deposits
